# Use and Costs of Supplemental Benefits in Medicare Advantage, 2017-2021

**DOI:** 10.1001/jamanetworkopen.2024.54699

**Published:** 2025-01-14

**Authors:** Christopher L. Cai, Sonia Iyengar, Steffie Woolhandler, David U. Himmelstein, Kavya Kannan, Lisa Simon

**Affiliations:** 1 Division of General Internal Medicine and Primary Care, Brigham and Women’s Hospital, Boston, Massachusetts; 2 Department of Medicine, Montefiore Einstein, Bronx, New York; 3 Department of Nutrition and Public Health, Hunter College, City University of New York, New York, New York; 4 Cambridge Health Alliance/Harvard Medical School, Cambridge, Massachusetts; 5 Department of Neuroscience, The University of Texas at Dallas

## Abstract

**Importance:**

Nearly all Medicare Advantage (MA) plans offer dental, vision, and hearing benefits not covered by traditional Medicare (TM). However, little is known about MA enrollees’ use of those benefits or how much they cost MA insurers or enrollees.

**Objective:**

To estimate use, out-of-pocket (OOP) spending, and insurer payments for dental, hearing, and vision services among Medicare beneficiaries.

**Design, Setting, and Participants:**

This cross-sectional analysis used pooled 2017-2021 Medical Expenditure Panel Survey (MEPS) and Medicare Current Beneficiary Survey (MCBS) data for MA and TM beneficiaries (excluding those also covered by Medicaid). The analysis was performed from September 10, 2023, to June 30, 2024.

**Exposures:**

MA compared with TM coverage.

**Main Outcomes and Measures:**

The main outcome was receipt of eye examinations, corrective lenses, hearing aids, optometry and dental visits, and MA and TM enrollees’ and insurers’ spending for such services. MEPS and MCBS data were weighted to be nationally representative.

**Results:**

We included 76 557 non–dually eligible Medicare beneficiaries, including 23 404 from the MEPS and 53 153 from the MCBS. Weighted demographic characteristics of MA and TM beneficiaries were similar (54.7% and 51.9% female; 39.8% and 35.2% older than 75 years, respectively). Only 54.2% (95% CI, 52.4%-55.9%) and 54.3% (95% CI 52.2%-56.3%) of MA beneficiaries were aware of having MA dental and vision coverage, respectively. MA enrollees were no more likely to receive eye examinations, hearing aids, or eyeglasses than TM enrollees. After adjustment for demographic differences, MA and TM enrollees paid OOP $205.86 (95% CI, $192.44-$219.27) and $226.12 (95% CI, $212.02-$240.23), respectively, for eyeglasses (MA − TM difference, −$20.27 [95% CI, −$33.77 to −$6.77] or −9.0% [95% CI, −14.9% to −3.0%]); $226.82 (95% CI, $202.24-$251.40) and $249.98 (95% CI, $226.22-$273.74) for dental visits, respectively (MA − TM difference, −$23.16 [95% CI, −$43.15 to −$3.17] or −9.3% [95% CI, −17.3% to −1.3%]); and no less for optometry visits or durable medical equipment (a proxy for hearing aids). Nationwide, MA plans’ annual spending on vision, dental services, and durable medical equipment totaled $3.9 billion (95% CI, $3.3-$4.4 billion), while enrollees spent OOP $9.2 billion (95% CI, $8.2-$10.2 billion) annually for these services and other private insurers covered $2.8 billion (95% CI, $2.7-$3.0 billion).

**Conclusions and Relevance:**

In this cross-sectional study of 2 nationally representative surveys, MA beneficiaries did not receive more supplemental services than TM beneficiaries, possibly because of cost-sharing and limited awareness of benefit coverage.

## Key Points

**Question:**

Does the use of dental, hearing, and vision care (ie, supplemental benefits or services) differ between non–dually eligible Medicare Advantage (MA) and traditional Medicare (TM) beneficiaries?

**Findings:**

In this cross-sectional study of 76 557 Medicare beneficiaries, more of those with MA than with TM had coverage of supplemental benefits, but spending and use were similar, and many MA beneficiaries were not aware of dental or vision coverage. The MA beneficiaries paid 9.0% and 9.3% less out of pocket for eyeglasses and dental visits, respectively, but no less for most other supplemental services.

**Meaning:**

These findings suggest that MA beneficiaries may not receive more supplemental services than TM beneficiaries possibly because of cost-sharing and limited awareness of benefit coverage.

## Teaser

This cross-sectional study estimates use, out-of-pocket spending, and insurer payments for dental, hearing, and vision services among Medicare Advantage and traditional Medicare beneficiaries.

## Introduction

Medicare Advantage (MA) plans enrolled 51% of Medicare beneficiaries in 2023.^1^ According to the Medicare Payment Advisory Commission, the federal government pays MA plans approximately 22% more than the cost of covering similar beneficiaries under traditional Medicare (TM),^2,3^ which is equivalent to overpayments of approximately $83 billion in 2024. MA plans might deploy a portion of overpayments to improve access to dental, hearing, or vision services (so-called supplemental benefits or services) not covered by TM, and some beneficiaries are attracted to MA plans’ advertisements of such benefits.^1^

Few studies have assessed MA enrollees’ use of supplemental services or the costs of such services to insurers or enrollees. A previous analysis found that more MA than TM enrollees had vision and dental coverage, although out-of-pocket (OOP) spending for vision and dental care was substantial for both groups.^4^ However, data processing errors in the 2016 Medicare Current Beneficiary Survey (MCBS) analyzed for that study compromised insurance coverage estimates.^5^ Moreover, the MCBS does not include visit-level data on cost-sharing or allow national estimates of total MA plan spending on supplemental benefits. While more than 95% of MA enrollees have access to supplemental (including dental) benefits,^6^ MA (vs TM) enrollees do not receive more dental care.^7,8^ In this study, we analyzed nationwide data on the use of hearing, dental, and vision care among TM and MA beneficiaries and OOP, and insurers’ expenditures for such services.

## Methods

For this cross-sectional study, the authors’ institutional review boards did not require ethics review and informed consent as the analyses were of deidentified, public-use data and considered to be non–human participant research. We followed the Strengthening the Reporting of Observational Studies in Epidemiology (STROBE) reporting guideline for cross-sectional studies.

We analyzed (pooled) 2017-2021 data from the MCBS to assess coverage of dental, hearing, and vision care and supplies (hereafter, supplemental benefits); the use of (and need for) those services; and beneficiaries’ problems in accessing dental care. Using the 2017-2021 Medical Expenditure Panel Survey (MEPS), we calculated insurers’ and patients’ expenditures for those services, adjusted to 2021 dollars using the Consumer Price Index. Further details of survey administration, outcome variables, covariates, and our methodology are provided in eTable 1 in Supplement 1. We excluded dually eligible (ie, covered by both Medicare and Medicaid) beneficiaries to avoid confounding by state-to-state variation in Medicaid coverage. The 2017-2021 MEPS lumped hearing aid expenditures with other durable medical equipment (DME) costs but reported them separately in 2016, which we used to calculate hearing aids’ share of DME that year and to assess the magnitude of our overestimation of hearing aid expenditures in 2017-2021.

### Statistical Analysis

We first compared the characteristics of MA and TM enrollees reported in the MCBS and MEPS. We then calculated the percentage of MA beneficiaries reporting MA coverage for eye examinations and dental care and the percentage of MA and TM beneficiaries reporting private dental or vision insurance. Next, we determined the percentage of MA and TM enrollees who received an eye examination, corrective lenses, or hearing aids both overall and among those reporting a need for them. Additionally, we assessed the proportion of MA and TM enrollees unable to obtain dental care because of cost or for any reason.

We then calculated total MA vs TM use, plan spending, and OOP beneficiary spending. Given the right skew of expenditure data, we used 2-part regressions for analyses of expenditures (eMethods in Supplement 1). We then calculated enrollees’ per-visit (or per-purchase) OOP spending for dental visits, optometry visits, and eyeglass purchases. We tabulated total DME costs, a proxy for hearing aid expenditures. In addition, we tabulated OOP spending and Medicare insurer spending for MA and TM enrollees nationally and per capita. To evaluate whether spending changed during the early years of the COVID-19 pandemic, we stratified results by year in a sensitivity analysis.

We report both unadjusted and adjusted differences between MA and TM enrollees (eMethods in Supplement 1). In multivariable models adjusted for demographic variables, we used the following categories: age (<65 years, 65-75 years, >75 years), sex (female, male), race and ethnicity (self-reported in the MCBS and grouped as Hispanic, non-Hispanic Black, non-Hispanic White, and other [including Asian American, American Indian or Alaska Native, Native Hawaiian or other Pacific Islander, multiracial, and not otherwise specified]), education (less than high school, high school, or vocational, technical, business, or more than high school), and income (≤100%, >100%-120%, >120%-135%, >135%-200%, >200% of the federal poverty level). Race and ethnicity were evaluated because MA plans have historically disproportionately enrolled racial and ethnic minority individuals who use less health care on average compared with their White counterparts. We opted not to adjust for indicators of severity of illness, which may be confounded by MA plans’ more intensive coding (and possibly ascertainment) of enrollees’ diagnoses, and use management, which may decrease hospitalizations and other health care use.

Analyses were performed from September 10, 2023, through June 30, 2024, using Stata, version 18.1 (StataCorp LLC), with survey weights that allow for national estimates and account for the complex survey design. Two-sided significance was set at *P* < .05 using χ^2^ test for categorical variables.

## Results

Our samples included 76 557 non–dually eligible Medicare beneficiaries, including 23 404 from the MEPS (mean [SE] annual weighted number, 47 664 111 [1 879 346]) and 53 153 from the MCBS (mean [SD] annual weighted number, 48 114 611 [2 551 586]). As expected in nationally representative surveys, the weighted demographic characteristics of the 2 samples were similar. MA (vs TM) enrollees were more often female (54.7% vs 51.9% [male, 45.3% vs 48.1%]) and more likely to be in older age categories (aged >75 years, 39.8% vs 35.2%), to be racial and ethnic minority individuals (Black, 9.6% vs 6.3%; Hispanic, 7.9% vs 4.7%; other race and ethnicity, 6.6% vs 6.4% [compared with White, 75.9% vs 82.6%]); to be in lower income categories (≤100% to ≤200% of the federal poverty level, 35.2% vs 24.0%); and to be in lower educational categories (less than high school, 11.4% vs 7.2%; high school or vocational, technical, or business school, 33.1% vs 29.6%) (Table). MA enrollment increased over time, while the number of TM enrollees remained relatively stable as total Medicare enrollment grew (eTable 2 in Supplement 1). Most metrics of inpatient, outpatient, and emergency service use were slightly lower in MA, though confidence intervals were wide (eTable 3 in Supplement 1).

**Table.** Characteristics of Medicare Advantage and Traditional Medicare Beneficiaries in the Medicare Current Beneficiary Survey, 2017-2021

Characteristic	No. of beneficiaries	Weighted, millions, No. (%)		*P* value^a^
		Medicare Advantage (n = 89 million)	Traditional Medicare (n = 151 million)	
Unweighted totals, No.	NA	21 604	31 549	NA
Sex (0 missing)				
Female	27 985	49 (54.7)	79 (51.9)	.002
Male	25 168	40 (45.3)	73 (48.1)	
Age group, y (0 missing)				
<65	4402	7 (8.1)	13 (8.3)	<.001
65-75	19 897	46 (52.1)	86 (56.5)	
>75	28 854	36 (39.8)	53 (35.2)	
Race and ethnicity (0 missing)				
Hispanic	3571	7 (7.9)	7 (4.7)	<.001
Non-Hispanic Black	3635	9 (9.6)	10 (6.3)	
Non-Hispanic White	43 045	68 (75.9)	130 (82.6)	
Other^b^	2902	6 (6.6)	10 (6.4)	
Family income, % of FPL (0 missing)				
≤100	3212	5 (6.0)	7 (4.6)	<.001
>100 and ≤120	2014	4 (4.2)	4 (2.8)	
>120 and ≤135	1868	3 (3.7)	4 (2.7)	
>135 and ≤200	9759	19 (21.3)	21 (13.9)	
>200	36 300	58 (64.8)	120 (76.1)	
Education (195 missing)				
Less than high school	5664	10 (11.4)	11 (7.2)	<.001
High school or vocational/technical/business	17 308	30 (33.1)	45 (29.6)	
More than high school	29 986	49 (55.1)	95 (63.0)	

Abbreviations: FPL, federal poverty level; NA, not applicable.

^a^ Tests for significance calculated using χ^2^ tests for categorical variables.

^b^ The Medicare Current Beneficiary Survey defines other race and ethnicity as Asian American, American Indian or Alaska Native, Native Hawaiian or Pacific Islander, multiracial, or not otherwise specified.

**Figure 1.**
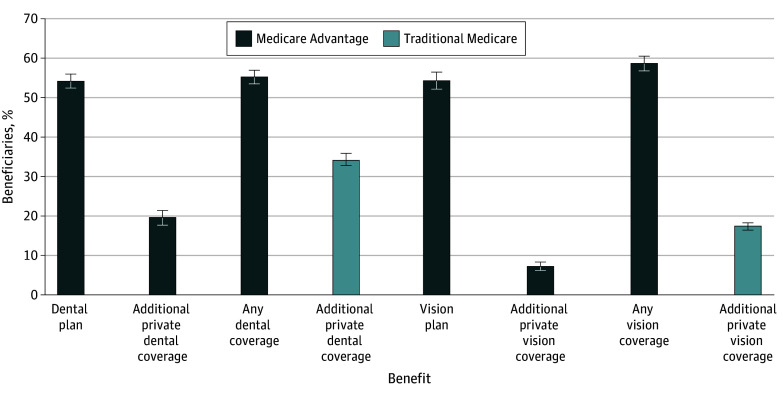
Self-Reported Coverage, Medicare Advantage vs Traditional Medicare Beneficiaries, Medicare Current Beneficiary Survey 2017-2021 Results are weighted to be nationally representative and unadjusted for differences in demographic characteristics. Full results, including adjusted differences, are provided in eTable 4 in Supplement 1. Error bars indicate 95% CIs.

Of MA enrollees, 54.2% (95% CI, 52.4%-55.9%) reported having dental coverage and 54.3% (95% CI, 52.2%-56.3%) vision (ie, eye examination) coverage from their MA plan (Figure 1), while 7.2% (95% CI, 6.0%-8.4%) of MA enrollees compared with 17.4% (95% CI, 16.5%-18.3%) of TM enrollees reported having other private vision coverage (Figure 1; eTable 4 in Supplement 1). Additional private dental coverage was reported by 19.5% (95% CI, 17.7%-21.4%) of MA enrollees and 34.2% (95% CI, 32.6%-35.9%) of TM enrollees. The share of MA beneficiaries reporting vision coverage through either MA or another private insurer (58.6%; 95% CI, 56.7%-60.5%) was only slightly higher than those reporting coverage through their MA plan alone.

**Figure 2.**
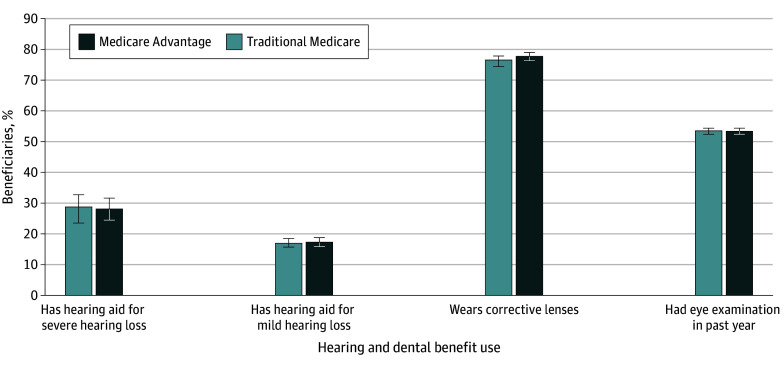
Medicare Advantage and Traditional Medicare Beneficiaries Reporting Use of Vision and Hearing Benefits, Medicare Current Beneficiary Survey 2017-2021 Results are weighted to be nationally representative and adjusted for sex, age, race and ethnicity, education, and income. Unadjusted differences were similar and are provided in eTable 4 in Supplement 1. Error bars indicate 95% CIs.

In adjusted analyses, MA enrollees compared with TM enrollees reported similar rates of trouble seeing (33.6% [95% CI, 32.5%-34.7%] vs 33.5% [95% CI, 32.8%-34.2%]; *P* = .89) and trouble hearing (42.7% [95% CI, 41.4%-44.0%] vs 43.0% [95% CI, 41.8%-44.2%]; *P* = .57) (unadjusted analyses provided in eTable 4 in Supplement 1). MA and TM enrollees, respectively, had virtually identical rates of wearing hearing aids overall (13.4% [95% CI, 12.7%-14.2%] vs 13.2% [95% CI, 12.5%-14.0%]; *P* = .67) and among those with mild hearing loss (17.3% [95% CI, 16.0%-18.6%] vs 16.9% [95% CI, 15.6%-18.2%]; *P* = .67) or severe hearing loss (28.1% [95% CI, 23.5%-32.7%] vs 28.8% [95% CI, 25.2%-32.3%]; *P* = .76) (Figure 2; eTable 4 in Supplement 1). Similarly, among individuals with trouble seeing, MA and TM enrollees, respectively, had similar rates of wearing corrective lenses (78.0% [95% CI, 76.2%-79.7%] vs 76.8% [95% CI, 75.5%-78.0%]; *P* = .26), and MA and TM enrollees overall had nearly identical rates of receipt of an eye examination in the past year (53.5% [95% CI, 52.6%-54.4%] vs 53.6% [95% CI, 52.8%-54.4%]; *P* = .85). In adjusted analyses, no differences were observed in delays in dental care overall or due to cost (difference in overall barriers to dental care: 0.6% [95% CI, −0.1% to 1.3%]; *P* = .12; difference in financial barriers to dental care: 0.0% [95% CI, −0.6% to 0.6%]; *P* = .98) (eTable 4 in Supplement 1). Select results varied little by survey year or in unadjusted analyses (eTable 5 in Supplement 1).

**Figure 3.**
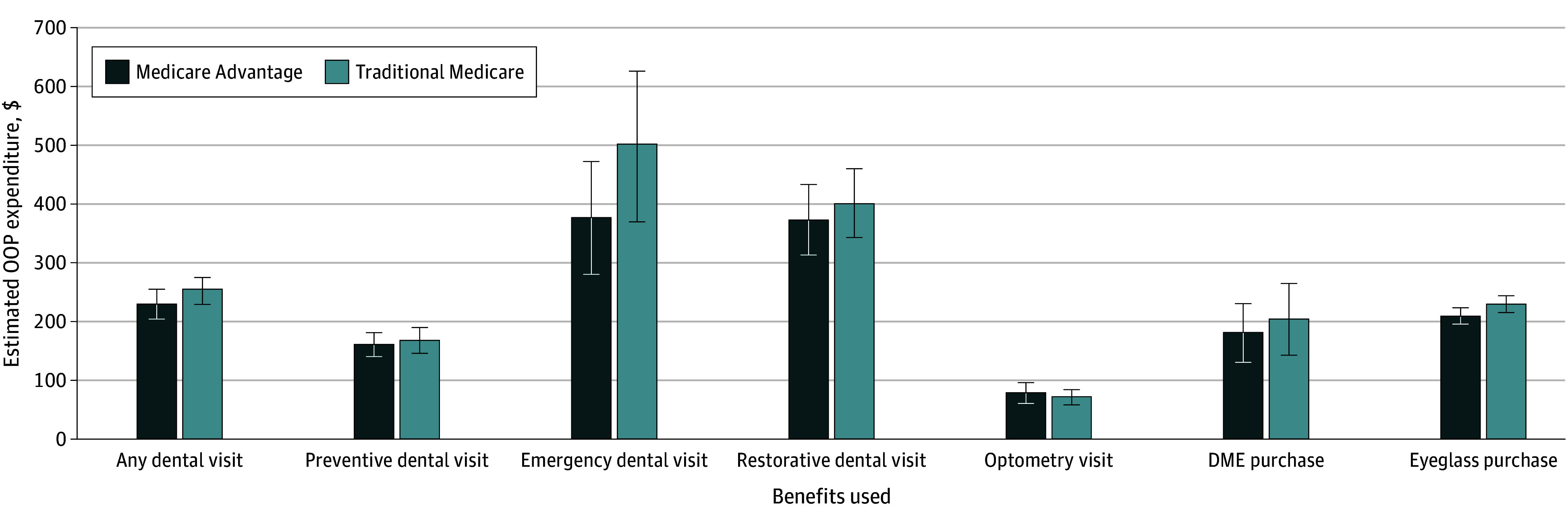
Out-of-Pocket (OOP) Expenditures for Dental, Vision, and Hearing Services Among Medicare Advantage and Traditional Medicare Enrollees, 2017-2021 Medical Expenditure Panel Surveys Estimated expenditures calculated as the marginal effects from 2-part models adjusted for differences in age, sex, region of US residence, education, income, and race and ethnicity. Medicare Advantage OOP expenditure per event was significantly different from traditional Medicare OOP expenditure at the *P* < .05 threshold. Dollars are adjusted to 2021 figures using the Consumer Price Index. Error bars represent 95% CIs. Full results are provided in the eFigure and eTables 6 and 7 in Supplement 1. DME indicates durable medical equipment.

Figure 3 displays MA and TM enrollees’ adjusted OOP expenditures per visit and purchase. The estimated mean OOP expenditures from 2-part models, and at various percentiles of OOP expenditures, are shown in eTables 6 and 7 in Supplement 1, respectively. In adjusted models, MA and TM enrollees paid $205.86 (95% CI, $192.44-$219.27) and $226.12 (95% CI, $212.02-$240.23), respectively, for eyeglasses (MA − TM difference, −$20.27 [95% CI, −$33.77 to −$6.77] or −9.0% [95% CI, −14.9% to −3.0%]). MA and TM beneficiaries paid $226.82 (95% CI, $202.24-$251.40) and $249.98 (95% CI, $226.22-$273.74) for dental visits, respectively (MA − TM difference −$23.16 [95% CI, −$43.15 to −$3.17] or −9.3% [95% CI, −17.3% to −1.3%]). MA and TM enrollees had no difference in OOP expenses for optometry visits or DME (Figure 3; eTable 6 in Supplement 1). The difference in MA vs TM dental OOP expenditures was driven by a difference in payments for emergency dental visits, with MA beneficiaries paying $374.93 (95% CI, $278.83-$471.03) vs $497.31 (95% CI, $369.33-$625.29) for TM beneficiaries or $122.38 less per visit (95% CI, $14.90-$229.86; *P* = .03). Adjusted differences in OOP payments between MA and TM did not vary significantly by year, though confidence intervals were wide (eFigure in Supplement 1).

Both MA and TM enrollees bore most of the costs for vision, dental, and hearing aid services OOP. Annual expenditures for these supplemental benefits for MA enrollees totaled $15.9 billion (95% CI, $14.2-$17.6 billion) across all payers (eTable 8 in Supplement 1). Enrollees paid $9.2 billion (95% CI, $8.2-$10.2 billion), representing 57.9% of the total or $499.52 (95% CI, $459.05-$539.99) per capita. TM enrollees paid 63.2% ($15.8 billion of the $25.0 billion total) of supplemental benefit expenditures OOP per year, equivalent to $573.02 (95% CI, $534.80-$611.23) per capita.

Nationally, MA plans spent $3.9 billion (95% CI, $3.3-$4.4 billion) annually ($202.51 per capita; 95% CI, $178.50-$226.53 per capita) on supplemental benefits, representing 24.5% of the total cost of supplemental benefits ($859.86 per capita; 95% CI, $801.58-$918.14 per capita) annually for their enrollees. In contrast, TM spent $1.6 billion (95% CI, $1.3-$1.9 billion) annually on supplemental benefits ($56.22 per capita; 95% CI, $45.83-$66.60 per capita), 6.4% of total expenditures for supplemental benefits ($902.38 per capita; 95% CI, $853.32-$951.43 per capita) for its enrollees. Non-MA private insurers accounted for the remainder of expenditures for both groups (other insurers for MA beneficiaries, $2.8 billion [95% CI, $2.7-$3.0 billion]; for TM beneficiaries, $7.6 billion [95% CI, $7.0-$8.1 billion]) (eTable 8 in Supplement 1). Sensitivity analyses that included all individuals who had MA during any survey round yielded similar results (eTables 9 and 10 in Supplement 1).

In 2016, hearing aids represented 52.4% of all MA plans’ spending on DME ($347 million of $662 million) (eTable 11 in Supplement 1). This finding suggests that our 2017-2021 estimates of MA spending on supplemental benefits, which used DME as a proxy for hearing aids, overestimated spending by approximately $520 million annually (48% of annual MA DME spending) and that actual MA annual spending on supplemental benefits likely totaled approximately $3.4 billion. Finally, spending on supplemental services did not significantly change during the COVID-19 pandemic (eTable 12 in Supplement 1).

## Discussion

Nearly all MA plans report coverage of supplemental benefits. However, in this national cross-sectional study, non–dually eligible MA beneficiaries did not receive more dental, vision, or hearing care than TM beneficiaries. This finding held even among subgroups with a specific medical need for such services, such as individuals with hearing loss who require hearing aids. MA enrollees’ limited use of supplementary services may reflect their lack of awareness of coverage for those services, as only one-half of MA beneficiaries reported dental or vision coverage, or barriers posed by cost-sharing imposed by plans, which covered only one-fourth of the total costs of dental, vision, and hearing services for MA beneficiaries.

MA plans spent approximately $200 annually per capita (approximately $145 more than TM) on supplemental benefits between 2017 and 2021, equivalent to 1% to 2% of total MA spending.^9,10,11^ During the same period, MA plans were paid, on average, $37.2 billion more annually than taxpayers would have spent had MA enrollees remained in TM (with overpayments expected to rise to $82 billion in 2024).^2^ Approximately 10% of those overpayments, $3.9 billion, was passed on to MA enrollees as payments for supplemental services. Some of the remainder was likely used to reduce premiums and copayments, although a recent study found that MA and TM enrollees incurred comparable overall OOP costs and experienced similar financial strains (including delaying care due to costs).^12^ While little is known about how MA plans spend the overpayments (as well as savings from MA enrollees’ lower care use), insurer overhead and profit appear to account for a large share: as much as 97% of overpayments between 2007 and 2024.^13^

Relatively few data have been available on the use or costs of supplemental benefits. A study using the 2016 MCBS data found that use and OOP payments for such benefits were similar for MA and TM beneficiaries.^5^ Our analysis includes more recent data, which corrected the data processing error in the 2016 MCBS, provides visit-level analyses of expenditures and use using the MEPS, and estimates national MA plan and TM expenditures on supplemental benefits. Our estimates of use in MA vs TM largely concur with a prior MCBS analysis.^4^ A KFF analysis using unverified self-reports from the 2018 MCBS found that among Medicare beneficiaries (including dually eligible individuals) using supplemental benefits, MA enrollees reported spending 20% to 23% less OOP for such services than TM beneficiaries.^14^ Our somewhat lower estimates encompass more recent data, exclude dually eligible individuals (who are known to have low OOP costs and represent a growing share of MA enrollees^15^), and may be less subject to recall bias. Finally, our results concur with prior work suggesting that MA beneficiaries had no higher use of preventive, restorative, or emergency dental visits.^7,16^

Several factors may underlie our findings. While more than 95% of beneficiaries are enrolled in MA plans with supplemental dental, hearing, and/or vision coverage,^6^ just over one-half of MA enrollees in our sample reported having such coverage, suggesting that many beneficiaries were unaware that their plan offered a dental and/or vision benefit. MA’s restrictions may also constrain enrollees’ use, as 52% of MA enrollees are in plans that require prior authorization for eye examinations; 55% for hearing examinations; 86% for comprehensive dental services; and 99% for DME, such as hearing aids.^3^ Most MA beneficiaries are also subject to annual caps or frequency limits on coverage for supplemental services,^14^ and limited provider networks may also constrain use in MA plans.^7,17,18^

We and others^15^ have found that individuals with lower income and from minoritized groups disproportionately enroll in MA. Hence, our unadjusted findings could be affected by the tendency of socioeconomically disadvantaged patients to use less care relative to their medical needs—regardless of their coverage—than others.^11,19,20,21^ Moreover, socioeconomically disadvantaged individuals often have particularly high levels of unmet dental, vision, and hearing needs.^19,20,21^ While our multivariable models adjusted for differences between the MA and TM enrollees with regard to income, education, and race and ethnicity, they did not account for TM enrollees’ higher rates of private coverage for supplemental benefits. Hence, MA coverage of those benefits may have compensated for the paucity of private coverage among enrollees, bringing the use of supplemental services for disadvantaged beneficiaries up to the level of the average TM enrollee. Alternatively, comparable rates of supplemental service use despite MA plans’ coverage of those benefits may reflect these individuals’ tendency to underuse care (eg, because of structural barriers, including racism), and MA plans’ selective enrollment of low-utilizing beneficiaries, so-called favorable selection.^1^

Not all use is desirable, and similar amounts of use may not indicate equivalent mixes of appropriate and inappropriate care. Nevertheless, even for preventive services, such as annual eye examinations, which are unlikely to represent overuse since they are indicated for all adults older than 65 years according to clinical guidelines,^7,22^ use differed little between TM and MA, with both groups having less-than-recommended use. Similarly, among enrollees with a clear need for services (eg, corrective lenses for vision loss, hearing aids for severe hearing loss), use was not higher in MA. Finally, while OOP payments for eyeglasses and a subgroup of dental visits were somewhat lower for MA (vs TM) enrollees, both groups incurred large OOP costs.

### Limitations

Our study has several limitations. First, our results should be interpreted as purely descriptive, as patients in MA may differ from those in TM on dimensions not captured in our adjusted analyses (ie, may be subject to residual confounding). Future studies might address this limitation by using propensity scores or similar methods. Second, the MEPS underestimates medical expenditures partly because it excludes institutionalized individuals, such as nursing home residents. Third, our estimates reflect the actual use of services, so we could not assess how many services were deferred because OOP costs were too high. Fourth, we lacked precise data on hearing aid costs in the MEPS, which, based on 2016 MEPS data, may represent approximately half of total DME costs, which we used as a proxy. Lack of these data probably led us to overestimate total MA expenditures on supplemental benefits by approximately $520 million annually. Fifth, MEPS does not query TM enrollees about supplemental dental coverage and lacks data on beneficiaries’ premium payments for MA and/or any supplemental private coverage, which may be higher among TM beneficiaries. In the MCBS, vision and dental coverage is self-reported and subject to recall bias, as is clinician specialty in the MEPS, which we used to identify optometry specialist visits. Sixth, we could not precisely determine whether MA or TM paid for care among individuals who switched between MA and TM midyear, although our sensitivity analysis addressing this issue indicates that any resulting error is small. Seventh, the public-use MEPS data do not provide granular geographic data, and MA penetration (and possibly the use and costs of supplemental services) varies by locality. We could not assess whether the Medigap coverage reported by some individuals who denied having supplemental benefit coverage included coverage for supplemental benefits, as do approximately 7% of Medigap plans.^23^

## Conclusions

Medicare beneficiaries have significant unmet dental, hearing, and vision needs,^24,25,26^ but this cross-sectional study found that while more MA enrollees have coverage of supplemental benefits, MA enrollment does not appear to be associated with more use of these services. Use might be increased if more MA beneficiaries knew of the benefits to which they are entitled, a problem that may be addressed by the Centers for Medicare & Medicaid Services’ proposal that starting in 2025, plans be required to send a midyear letter informing their enrollees of any unused benefits.^27^ However, broader measures are needed to expand supplemental benefit coverage and use for all Medicare beneficiaries, who collectively incurred $25 billion annually in OOP costs for vision, hearing, and dental care during the study period. Funding for such an expansion, and to reduce cost-sharing for services covered under Parts A and B of TM, might be garnered by reducing or eliminating overpayments to MA insurers.^28^

## Notes

Supplement 1. **eTable 1.** Medicare Current Beneficiary Survey (MCBS) Variables Used in Analysis
eMethods.
**eTable 2.** Numbers of Medicare Advantage and Traditional Medicare Enrollees by Year, 2017-2021 **eTable 3.** Summary Annual Utilization Statistics, Medicare Advantage and Traditional Medicare, 2017-2021 **eTable 4.** Vision, Hearing, and Dental Benefits, Needs and Utilization Among Medicare Advantage and Traditional Medicare Beneficiaries, MCBS 2017-2021 **eTable 5.** Unadjusted Utilization of Supplemental Benefits by Year, MCBS 2017-2021 **eTable 6.** Predicted Mean Out-of-Pocket Expenditures for Visit or Purchase, MA vs TM, 2017-2021 **eTable 7.** Annual Out-of-Pocket Costs per Event at 5th, 25th, 50th, 75th, and 95th Percentiles of Expenditures, 2021 Dollars, MEPS 2017-2021 **eFigure.** Unadjusted Differences in Out-of-Pocket Payment per Event by Year, Medicare Advantage Minus Traditional Medicare, MEPS 2017-2021 **eTable 8.** Annual Spending on Vision, Dental, and Durable Medical Equipment by Patients, Medicare Insurers, and non-MA Private Insurers, MEPS 2017-2021, 2021 Dollars **eTable 9.** Number of Individuals Included in Each Definition of Medicare Advantage or Traditional Medicare Enrollment, MEPS 2017-2021 **eTable 10.** Total Annual Spending by MA Plans, Traditional Medicare, and Private Insurers Other Than MA, Sensitivity Analysis **eTable 11.** Spending for Hearing Aids and for Durable Medical Equipment by Patients and Medicare Insurers, 2016 MEPS **eTable 12.** Total Spending Nationally by Beneficiaries, MA Plans, and Traditional Medicare on Supplemental Benefits by Year, MEPS 2017-2021
eReferences

Supplement 2.
Data Sharing Statement
