# Supplementary material for: Use and Costs of Supplemental Benefits in Medicare Advantage, 2017-2021
Source: JAMA Netw Open. 2025 Jan 14;8(1):e2454699. doi: 10.1001/jamanetworkopen.2024.54699 (PMC11733699; doi:10.1001/jamanetworkopen.2024.54699)
Supplement: Supplement 1. — eTable 1. Medicare Current Beneficiary Survey (MCBS) Variables Used in Analysis eMethods. eTable 2. Numbers of Medicare Advantage and Traditional Medicare Enrollees by Year, 2017-2021 eTable 3. Summary Annual Utilization Statistics, Medicare Advantage and Traditional Medicare, 2017-2021 eTable 4. Vision, Hearing, and Dental Benefits, Needs and Utilization Among Medicare Advantage and Traditional Medicare Beneficiaries, MCBS 2017-2021 eTable 5. Unadjusted Utilization of Supplemental Benefits by Year, MCBS 2017-2021 eTable 6. Predicted Mean Out-of-Pocket Expenditures for Visit or Purchase, MA vs TM, 2017-2021 eTable 7. Annual Out-of-Pocket Costs per Event at 5th, 25th, 50th, 75th, and 95th Percentiles of Expenditures, 2021 Dollars, MEPS 2017-2021 eFigure. Unadjusted Differences in Out-of-Pocket Payment per Event by Year, Medicare Advantage Minus Traditional Medicare, MEPS 2017-2021 eTable 8. Annual Spending on Vision, Dental, and Durable Medical Equipment by Patients, Medicare Insurers, and non-MA Private Insurers, MEPS 2017-2021, 2021 Dollars eTable 9. Number of Individuals Included in Each Definition of Medicare Advantage or Traditional Medicare Enrollment, MEPS 2017-2021 eTable 10. Total Annual Spending by MA Plans, Traditional Medicare, and Private Insurers Other Than MA, Sensitivity Analysis eTable 11. Spending for Hearing Aids and for Durable Medical Equipment by Patients and Medicare Insurers, 2016 MEPS eTable 12. Total Spending Nationally by Beneficiaries, MA Plans, and Traditional Medicare on Supplemental Benefits by Year, MEPS 2017-2021 eReferences [file jamanetwopen-e2454699-s001.pdf]

Supplemental Online Content

Cai CL, Iyengar S, Woolhandler S, Himmelstein D, Kannan K, Simon L. Use and costs of supplemental benefits in Medicare Advantage, 2017-2021. *JAMA Netw Open*. 2025;8(1):e2454699. doi:10.1001/jamanetworkopen.2024.54699

eTable 1. Medicare Current Beneficiary Survey (MCBS) Variables Used in Analysis

eMethods

eTable 2. Numbers of Medicare Advantage and Traditional Medicare Enrollees by Year, 2017-2021

eTable 3. Summary Annual Utilization Statistics, Medicare Advantage and Traditional Medicare, 2017-2021

eTable 4. Vision, Hearing, and Dental Benefits, Needs and Utilization Among Medicare Advantage and Traditional Medicare Beneficiaries, MCBS 2017-2021

eTable 5. Unadjusted Utilization of Supplemental Benefits by Year, MCBS 2017-2021

eTable 6. Predicted Mean Out-of-Pocket Expenditures for Visit or Purchase, MA vs TM, 2017-2021

eTable 7. Annual Out-of-Pocket Costs per Event at 5th, 25th, 50th, 75th, and 95th Percentiles of Expenditures, 2021 Dollars, MEPS 2017-2021

eFigure. Unadjusted Differences in Out-of-Pocket Payment per Event by Year, Medicare Advantage Minus Traditional Medicare, MEPS 2017-2021

eTable 8. Annual Spending on Vision, Dental, and Durable Medical Equipment by Patients, Medicare Insurers, and non-MA Private Insurers, MEPS 2017-2021, 2021 Dollars

eTable 9. Number of Individuals Included in Each Definition of Medicare Advantage or Traditional Medicare Enrollment, MEPS 2017-2021

eTable 10. Total Annual Spending by MA Plans, Traditional Medicare, and Private Insurers Other Than MA, Sensitivity Analysis

eTable 11. Spending for Hearing Aids and for Durable Medical Equipment by Patients and Medicare Insurers, 2016 MEPS

eTable 12. Total Spending Nationally by Beneficiaries, MA Plans, and Traditional Medicare on Supplemental Benefits by Year, MEPS 2017-2021

eReferences

This supplemental material has been provided by the authors to give readers additional information about their work.

© 2025 Cai CL et al. *JAMA Network Open.*

eTable 1. Medicare Current Beneficiary Survey (MCBS) Variables Used in Analysis

Outcome Variable In our Analysis	Variable Description in MCBS Codebook	MCBS Variable	Notes
MA only- report dental coverage by MA	Mcare Adv plan covers eye exams ?	ma_madveye	
MA only- report eye exam coverage by MA	Mcare Adv plan covers dental ?	ma_madvdent	
Private Vision plan	Private plan covers vision ?	ins_privvis	
Private Dental plan	Private plan covers dental ?	ins_privds	
Trouble Seeing	Description of SP's Vision	hlt_ectroub	Answers 2 (“a little trouble seeing”) and 3 (“a lot of trouble seeing”) were combined in binary variable for our analysis (“any trouble seeing”).
Eye Exam last year	Has the survey respondent had Eye Exam since Ref Date ?	hlt_edocexam	Reference date refers to whether a survey respondent had an exam within the last survey year.
Wears Corrective Lens	Does the Respondent Wear Eyeglasses/Contact Lenses?	hlt_echelp	
If trouble seeing, wearing corrective lens	N/A	N/A	Combination of variables above
Trouble hearing	Description of SP's hearing	hlt_hctroub	Mild hearing was defined as responding yes to "a little trouble hearing" and severe hearing as yes to "a lot of trouble hearing"
Wearing a Hearing Aid	Does the survey respondent use a hearing aid ?	hlt_hchelp	
Hearing Aid Among Those With Mild Hearing Loss	N/A	n/a	Combination of variables above
Hearing Aid Among Those with Severe Hearing Loss	N/A	n/a	Combination of variables above
Delayed Dental Care	Ever a time SP couldn’t get dental care?	acw_dvneed	
Delayed Dental Care Due to Cost	Couldn't get dental Care Due to Cost?	acw_dvndcost	

Results represent variables utilized in our analysis as well as description of questions presented to survey participants.

© 2025 Cai CL et al. *JAMA Network Open.*

eMethods

Survey Background

The two nationally representative surveys we analyzed provide complementary information on supplemental service coverage, utilization and expenditures. Hearing aid utilization and reliable estimates of dental coverage are not available in the 2017 MEPS and onward but are available in the MCBS. The MCBS lacks visit level expenditure information which is available in the MEPS. Both of these surveys encompass non-institutionalized, civilian Medicare beneficiaries. All data are self-reported in the MCBS (variable specification and the wording of MCBS questions appear in supplement eTable 1). In the MEPS, respondents' reports of expenditures and use are verified with providers when possible.

Expenditures in the MEPS are collected via surveys of participants and medical providers. For optometry visits, out-of-pocket expenditures are collected from interviews with household survey participants with a subset confirmed via surveys of the corresponding health care providers. Glasses purchases, durable medical equipment purchases and dental visits were also collected by survey, but none of these expenditures were validated by provider surveys. Data is edited for outliers and missingness. We communicated with survey administrators at the AHRQ who confirmed that Medicare Advantage expenditures are classified as "Medicare" rather than "private insurance" in the MEPS. Older studies suggest these estimates of expenditures may underestimate total expenses by 10-15% although out-of-pocket estimates are likely more accurate. [1,2]

Variable Definitions

For MEPS analyses, we defined Medicare Advantage enrollment by insurance held at the end of the year. In sensitivity analyses, we used an alternative definition which included anyone who had MA during any survey round during the year. In multivariable models adjusted for demographic variables, we used the following categories: age (<65 years, 65-76, 75 and older), sex (male, female), self-described race/ethnicity (Non-Hispanic white, Non-Hispanic Black, Hispanic, Other), education (less than high school, high school or vocational/technical/business/more than high school), and income (less than 100% of the federal poverty line [FPL], >100%-120%,>120% -135%, >135%-200%, > 200%). Race and ethnicity were self-reported in the MCBS. The MCBS defines "Other" race/ethnicity as including Asian-Americans, Native Hawaiian or Pacific Islander, American Indian or Alaskan Native, multi-racial individuals, and not otherwise specified backgrounds. For the education variable in the MEPS, we omitted individuals less than 25 years of age as in prior analyses.[3] The MCBS does not have a continuous variable for age

MEPS separately reported expenditures for hearing aids and other durable medical equipment in 2016, but lumped them into a single category in subsequent years. Hence, we used the 2016 MEPS to calculate the share of durable medical equipment expenditures attributable to hearing aids that year, which we used to assess the magnitude of our overestimation of hearing aid expenditures in 2017-2021.

Analysis

When assessing differences in supplemental benefit utilization using the MCBS, we adjusted for age, sex, race/ethnicity, education, and income using the demographic variables above. We performed multivariable ordinary least-squares linear regressions adjusted for demographic factors.

In analyzing per-visit OOP expenditures in the MEPS, for which many beneficiaries had zero expenditures and a few had high expenditures, we used a two-part model with a log link and gamma distribution, i.e. a probit model to estimate the probability of any OOP spending and a generalized linear model for individuals with expenditures (following prior analyses of the MEPS).[4,5] We then calculated expenditures using marginal effects from both parts of the model, first unadjusted and then adjusted for sex, age, race/ethnicity, income, region, and education. In sensitivity analyses for expenditures, we stratified spending per supplemental benefit event (Dental Visit, Optometry Visit, Durable Medical Equipment Purchase, Glasses Purchase) by year, given that the composition of MA vs TM plans have changed over time. We plotted the marginal effects of Medicare Advantage on OOP costs by year.

© 2025 Cai CL et al. *JAMA Network Open.*

eTable 2. Numbers of Medicare Advantage and Traditional Medicare Enrollees by Year, MEPS 2017-2021.

Year	MA, no Medicaid (millions)	95% confidence interval		TM, no Medicaid (millions)	95% confidence interval		MA+ Medicaid (millions)	95% confidence interval		TM + Medicaid (millions)	95% confidence interval	
2017	17.1	15.6	18.6	29.0	26.6	31.3	3.2	2.8	3.7	5.3	4.7	6.0
2018	18.5	16.3	20.7	29.0	25.9	32.2	3.5	2.9	4.1	5.5	4.6	6.3
2019	18.1	16.3	19.9	29.4	26.3	32.4	3.2	2.7	3.7	5.2	4.4	6.0
2020	20.6	18.4	22.7	28.9	25.9	31.8	3.8	3.2	4.4	4.8	4.0	5.5
2021	21.2	19.2	23.2	26.6	24.1	29.2	4.9	4.2	5.5	5.1	4.3	5.8

TM=Traditional Medicare. MA=Medicare Advantage.

© 2025 Cai CL et al. *JAMA Network Open.*

eTable 3. Summary Annual Utilization Statistics, Medicare Advantage and Traditional Medicare, 2017-2021

	**Medicare Advantage (n=9,560 unweighted, 2017-2021,95,500,000 weighted)**			**Traditional Medicare (n=13,844 unweighted, 2017-2021, 143,000,000 weighted)**				
	**Outcome**	**95% CI**		**Outcome**	**95% CI**		**MA minus TM unadjusted difference**	**P-value**
Any inpatient discharge	13.5%	12.6%	14.4%	14.4%	13.6%	15.2%	-0.9%	0.12
Mean discharges per year	0.19	0.17	0.20	0.21	0.19	0.22	-0.02	0.052
Any outpatient visit	91.5%	90.7%	92.2%	90.3%	89.5%	91.1%	1.2%	0.03
Mean outpatient visits per year	7.1	6.9	7.3	7.5	7.3	7.7	-0.4	0.01
Any emergency visit	19.9%	19.0%	20.9%	21.0%	20.0%	21.9%	-1.0%	0.12
Mean emergency visits per year	0.30	0.28	0.31	0.32	0.30	0.34	-0.21	0.11
Any prescription medication	92.1%	91.2%	93.0%	91.5%	90.8%	92.2%	0.6%	0.28
Mean total prescription medication refills per year	21.9	21.0	22.8	22.2	21.4	22.9	-0.3	0.58

Unadjusted difference reflects the difference in outcomes .TM=Traditional Medicare. MA=Medicare Advantage.

© 2025 Cai CL et al. *JAMA Network Open.*

eTable 4. Vision, Hearing, and Dental Benefits, Needs and Utilization Among Medicare Advantage and Traditional Medicare Beneficiaries, MCBS 2017-2021

		MA Unadjusted %	95% CI		TM Unadjusted%	95% CI		Unadjusted Difference %	95% CI		P-value	Adjusted Difference %[a]	95% CI		P-value
Reported Coverage	Dental coverage by MA	54.2	52.4	55.9	[redacted]										
	Eye exam coverage by MA	54.3	52.2	56.3											
	Private vision plan	7.2	6.0	8.4	17.4	16.5	18.3	-10.2	-11.7	-8.7	<0.001	-9.1	-10.5	-7.7	<0.001
	Private dental plan	19.5	17.7	21.4	34.2	32.6	35.9	-14.7	-17.1	-12.3	<0.001	-12.0	-14.2	-9.8	<0.001
Vision	Trouble seeing	32.2	31.1	33.3	31.0	30.3	31.8	1.2	0.0	2.3	0.047	0.08	-1.0	1.2	0.89
	Eye exam last year	55.5	54.5	56.5	56.5	55.7	57.3	-1.0	-2.3	0.3	0.13	-0.1	-1.3	1.0	0.85
	Wears corrective lens	84.4	83.4	85.4	84.1	83.4	84.9	0.2	-0.9	1.4	0.68	0.9	-0.3	2.0	0.13
	Wears corrective lens. Among those with vision loss	78.5	76.8	80.3	77.8	76.5	79.0	0.7	-1.4	2.9	0.49	1.2	-0.9	3.3	0.26
Hearing	Trouble hearing	43.4	42.3	44.6	44.0	42.8	45.1	-0.6	-1.8	0.7	0.38	-0.3	-1.6	0.9	0.57
	Wearing a hearing aid	14.7	13.8	15.5	14.9	14.1	15.6	-0.2	-1.2	0.8	0.72	0.2	-0.7	1.1	0.67
	Hearing aid among those with mild hearing loss	20.0	18.7	21.3	20.3	19.0	21.6	-0.3	-2.0	1.3	0.71	0.3	-1.3	1.9	0.67
	Hearing aid among those with severe hearing loss	33.0	28.7	37.3	34.0	30.9	37.1	-1.0	-5.5	3.5	0.66	-0.7	-5.2	3.8	0.76
Dental	Delayed dental care	10.2	9.5	10.9	8.5	7.8	9.1	1.7	0.9	2.5	<0.001	0.6	-0.1	1.3	0.12
	Delayed dental care due to cost	5.9	5.3	6.4	4.9	4.3	5.4	1.0	0.3	1.8	0.01	0.0	-0.6	0.6	0.98

P-values calculated as adjusted and unadjusted two-sided t tests. [a] Adjusted for sex, age, race/ethnicity, education, income

© 2025 Cai CL et al. *JAMA Network Open.*

eTable 5. Unadjusted Utilization of Supplemental Benefits by Year, MCBS 2017-2021

	Difference in likelihood of annual eye exam	95% CI		Difference in likelihood of wearing corrective lenses	95% CI	
2017	-1.6%	-4.4%	1.2%	0.6%	-1.2%	2.5%
2018	-0.2%	-2.7%	2.4%	0.9%	-0.6%	2.4%
2019	-1.2%	-3.5%	1.0%	0.6%	-1.3%	2.6%
2020	-0.5%	-3.0%	1.9%	-0.3%	-2.4%	1.7%
2021	-0.9%	-4.0%	2.1%	0.5%	-1.4%	2.3%
	**Difference in likelihood of hearing aid use**	**95% CI**		**Difference in likelihood of delaying dental care**	**95% CI**	
2017	-0.5%	-2.0%	1.0%	1.2%	-0.3%	2.7%
2018	-0.1%	-1.7%	1.6%	1.9%	0.3%	3.4%
2019	-0.6%	-2.2%	1.0%	1.4%	-0.1%	2.9%
2020	-0.2%	-1.6%	1.3%	1.5%	-0.1%	3.1%
2021	0.0%	-1.4%	1.5%	2.4%	1.1%	3.6%

All differences presented as (% in Medicare Advantage)-(% in Traditional Medicare). All Results are unadjusted.

© 2025 Cai CL et al. *JAMA Network Open.*

eTable 6. Predicted Mean Out-of-Pocket Expenditures for Visit or Purchase, MA vs. TM, MEPS 2017-2021.

	MA	TM	Marginal Effects (MA minus TM)			
	**Unadjusted Predicted Expenditure**	**Unadjusted Predicted Expenditure**	**Unadjusted Difference**	**95% CI**		**P-Value**
Any Dental Visit	251.42	272.08	-20.66	-43.30	1.98	0.07
Preventative Dental Visit	170.57	173.66	-3.08	-21.13	14.97	0.74
Emergency Dental Visit	514.24	695.47	-181.24	-329.82	-32.65	<0.001
Restorative Dental Visit	457.37	495.60	-38.23	-97.86	21.41	0.21
Optometry Visit	71.85	66.98	4.86	-15.64	25.37	0.64
Durable Medical Equipment Purchase	441.58	546.53	-104.95	-269.66	59.76	0.21
Glasses Purchase	242.74	277.03	-34.28	-50.93	-17.64	<0.001
	**Adjusted Predicted Expenditure**	**Adjusted Predicted Expenditure**	**Adjusted Difference**	**95% CI**		**P-Value**
Any Dental Visit	226.82	249.98	-23.16	-43.15	-3.17	0.02
Preventive Dental Visit	157.48	164.62	-7.13	-23.90	9.63	0.40
Emergency Dental Visit	374.93	497.31	-122.38	-229.86	-14.90	0.03
Restorative Dental Visit	370.70	400.32	-29.62	-75.96	16.72	0.21
Optometry Visit	74.50	68.78	5.72	-11.16	22.60	0.51
Durable Medical Equipment Purchase	178.58	201.31	-22.72	-82.78	37.33	0.46
Glasses Purchase	205.86	226.12	-20.27	-33.77	-6.77	<0.001

Marginal adjusted and unadjusted predicted out of pocket expenditure for a visit or purchase using two-part model. Results adjusted to 2021 dollars and adjusted for age, sex, income, region, education, race/ethnicity. MA= Medicare Advantage. TM= Traditional Medicare.

© 2025 Cai CL et al. *JAMA Network Open.*

eTable 7. Annual Out-of-Pocket Costs Per Event at 5th, 25th, 50th, 75th, and 95th Percentiles of Expenditures, 2021 Dollars, MEPS 2017-2021

	MA					TM				
**Percentile**	**5%**	**25%**	**50%**	**75%**	**95%**	**5%**	**25%**	**50%**	**75%**	**95%**
Any Dental Visit	$0	$0	$53	$178	$1,200	$0	$0	$69	$185	$532
Preventative Dental Visit	$0	$0	$55	$150	$580	$0	$0	$70	$155	$500
Emergency Dental Visit	$0	$0	$104	$391	$2,630	$0	$0	$160	$575	$3,400
Restorative Dental Visit	$0	$0	$68	$432	$2,000	$0	$0	$77	$430	$2,083
Optometry Visit	$0	$0	$20	$50	$355	$0	$0	$20	$65	$334
Durable Medical Equipment Purchase	$0	$8	$43	$155	$2,400	$0	$0	$39	$160	$3,100
Glasses Purchase	$0	$83	$200	$350	$650	$0	$100	$220	$398	$763.00

MA= Medicare Advantage. TM=Traditional Medicare. Percentiles represent percentile of out-of-pocket expenditures per visit or purchase. Results are rounded to the nearest dollar.

© 2025 Cai CL et al. *JAMA Network Open.*

eFigure. Unadjusted Difference in Out-of-Pocket Payment per Event by Year, Medicare Advantage Minus Traditional Medicare, MEPS 2017-2021

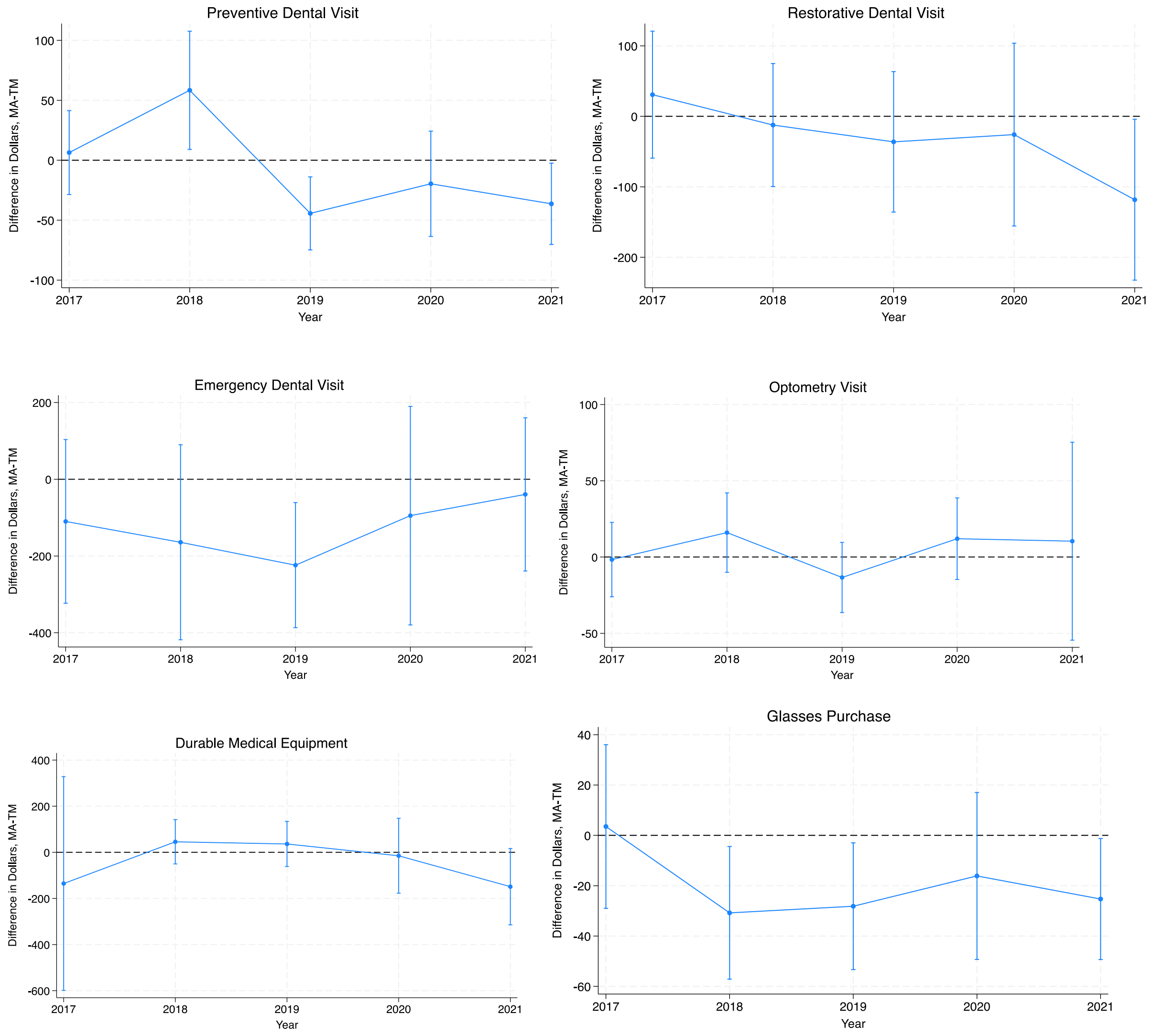

MA= Medicare Advantage. TM=Traditional Medicare. Results represent the average marginal effect of Medicare Advantage on the supplemental benefit (calculated as Medicare Advantage-Traditional Medicare).

© 2025 Cai CL et al. *JAMA Network Open.*

eTable 8. Annual Spending on Vision, Dental, and Durable Medical Equipment by Patients, Medicare Insurers, and non-MA Private Insurers, MEPS 2017-2021, 2021 Dollars

	Medicare Advantage (n=9,560 unweighted, total 2017-2021 95,500,000 weighted)			Traditional Medicare (n=13,844 unweighted, total 2017-2021 143,000,000 weighted)		
	$ Billions	95% CI		$ Billions	95% CI	
Glasses-patient OOP	1.3	1.1	1.4	2.0	1.8	2.2
Glasses-Medicare	0.3	0.3	0.4	0.1	0.0	0.1
Glasses-All Payors	1.8	1.6	2.0	2.7	2.4	2.9
DME-patient OOP	1.2	0.9	1.5	2.2	1.6	2.8
DME-Medicare	1.1	0.8	1.4	1.1	0.8	1.4
DME-All Payors	2.7	2.1	3.3	4.2	3.4	5.0
Dental-patient OOP	6.4	5.7	7.2	11.2	10.0	12.5
Dental-Medicare	2.1	1.8	2.5	0.2	0.2	0.3
Dental-All Payors	10.7	9.5	11.9	17.0	15.2	18.8
Optometry-patient OOP	0.2	0.2	0.3	0.4	0.3	0.4
Optometry Medicare	0.3	0.3	0.4	0.2	0.1	0.2
Optometry-All Payors	0.7	0.6	0.8	1.1	1.0	1.2
Total annual spending-patient OOP	9.2	8.2	10.2	15.8	14.1	17.5
Total Annual spending - Medicare	3.9	3.3	4.4	1.6	1.3	1.9
Total Annual Spending-Other Insurers	2.8	2.7	3.0	7.6	7.0	8.1
Total Annual Spending-all Payors	15.9	14.2	17.6	25.0	22.4	27.5

Patient OOP represents total annual spending by all beneficiaries on that benefit. DME= Durable Medical Equipment. Medicare= Medicare Advantage or Traditional Medicare. All results adjusted to $2021 dollars

© 2025 Cai CL et al. *JAMA Network Open.*

eTable 9. Number of Individuals Included in Each Definition of Medicare Advantage or Traditional Medicare Enrollment, MEPS 2017-2021

	**Primary Analysis- Defined by Insurance Status at end of survey year**		
	**No Medicaid**	**Medicaid**	**Total**
Traditional Medicare	13,844	3,376	17,220
Medicare Advantage	9,560	2,476	12,036
Total	23,404	5852	29,256
	Alternative Definition of MA- at any survey round in year		
Traditional Medicare	10,925	2,405	13,330
Medicare Advantage, alternative definition	13,189	3,707	16,896
Total	24,114	6,112	30,226
	Alternative Definition of TM- at any survey round in year		
Traditional Medicare, alternative definition	18,363	4,912	23,275
Medicare Advantage, alternative definition	6,205	1,424	7,629
Total	24,568	6,336	30,904

Results represent alternative definitions of MA or TM coverage in any of the MEPS survey rounds. MA=Medicare Advantage. TM=Traditional Medicare

© 2025 Cai CL et al. *JAMA Network Open.*

eTable 10. Total Annual Spending by MA Plans, Traditional Medicare, and Private Insurers Other than MA , Sensitivity Analysis

	Medicare Advantage (n=13,189 unweighted, 132,000,000 weighted, 2017-2021)			Traditional Medicare (n=18,363 unweighted, weighted 188,000,000, 2017-2021 period)		
	$ Billions	95% CI		$ Billions	95% CI	
Glasses-patient OOP	1.7	1.6	1.9	2.5	2.3	2.8
Glasses-Medicare	0.4	0.3	0.4	0.1	0.1	0.1
Glasses-Total	2.4	2.2	2.7	3.4	3.1	3.8
DME-patient OOP	1.8	1.4	2.2	2.7	2.1	3.3
DME-Medicare	1.4	1.0	1.8	1.6	1.3	2.0
DME-Total	3.8	3.1	4.5	5.4	4.5	6.4
Dental-patient OOP	8.8	7.8	9.8	14.0	12.4	15.5
Dental-Medicare	2.4	2.0	2.8	0.6	0.5	0.7
Dental-Total	14.2	12.7	15.8	21.2	19.0	23.4
Optometry-patient OOP	0.3	0.2	0.4	0.4	0.4	0.5
Optometry Medicare	0.4	0.3	0.4	0.3	0.2	0.3
Optometry-Total	0.9	0.8	1.1	1.3	1.2	1.5
Total annual spending-patient OOP	12.7	11.3	14.0	19.6	17.6	21.7
Total Annual spending - Medicare	4.6	3.9	5.2	2.6	2.2	3.1
Total Annual Spending-Other Insurers	4.1	4	4.4	9.2	8.5	9.7
Total Annual Spending-all Payors	21.4	19.2	23.6	31.4	28.3	34.5

Medicare Advantage Beneficiaries defined as any beneficiary who had MA in any of three survey rounds; TM Beneficiaries defined as any beneficiary who had TM in any of three survey rounds. Patient OOP represents total annual spending by all beneficiaries on that benefit. DME= Durable Medical Equipment. Medicare= Medicare Advantage or Traditional Medicare. All results adjusted to $2021 dollars

© 2025 Cai CL et al. *JAMA Network Open.*

eTable 11. Spending for Hearing Aids and for Durable Medical Equipment by Patients and Medicare Insurers, 2016 MEPS

	Medicare Advantage			Traditional Medicare		
	**$ Millions**	**95% CI**		**$ Millions**	**95% CI**	
Medicare Payments for Hearing Aids	347	72	621	9	-9	27
Out of Pocket Payments for Hearing Aids	1230	521	1940	1630	989	2280
Medicare Payments for all DME	662	377	948	394	226	561
Out of Pocket Payments for all DME	1430	709	2160	2310	1490	3120

DME = durable medical equipment

© 2025 Cai CL et al. *JAMA Network Open.*

eTable 12. Total Spending Nationally by Beneficiaries, MA Plans, and Traditional Medicare on Supplemental Benefits by Year, MEPS 2017-2021

	MA beneficiary out-of-pocket ($ billions)	95% CI		TM beneficiary out-of-pocket ($ billions)	95% CI	
2017	6.7	5.6	7.8	12.8	10.5	15.2
2018	10.3	8.4	12.3	16.0	13.3	18.6
2019	8.5	7.1	9.8	16.6	14.4	18.8
2020	9.0	7.1	10.9	14.6	12.1	17.1
2021	11.5	9.5	13.5	18.8	15.6	22.0
	**MA Plan payment ($ billions)**	**95% CI**		**TM Plan payment ($ billions)**	**95% CI**	
2017	2.9	2.1	3.8	0.8	0.5	1.0
2018	3.5	2.7	4.4	2.4	1.5	3.4
2019	4.1	2.9	5.3	1.3	0.9	1.8
2020	4.1	3.0	5.3	1.5	1.0	2.1
2021	4.7	3.8	5.5	2.0	1.3	2.7

Self-pay=total national spending by beneficiaries on supplemental vision, hearing or dental benefits. MA= Medicare Advantage. TM=Traditional Medicare

© 2025 Cai CL et al. *JAMA Network Open.*

eReferences

1. Zuvekas SH, Olin GL. Accuracy of Medicare Expenditures in the Medical Expenditure Panel Survey. *Inq J Health Care Organ Provis Financ*. 2009;46(1):92-108. doi:10.5034/inquiryjrnl_46.01.92

2. Machlin SR, Cohen JW, Zuvekas SH, Thorpe JM. Accuracy Of Household Reported Payments For Physician Visits In The 1996 Medical Expenditure Panel Survey. *Proceedings of the American Statistical Association of the Section on Survey Research Methods*. Published online 1999.

3. Cai C, Gaffney A, McGregor A, et al. Racial and ethnic disparities in outpatient visit rates across 29 specialties. *JAMA Intern Med*. 2021;181(11):1525-1527.

4. Simon L, Cai C. Dental Use and Spending in Medicare Advantage and Traditional Medicare, 2010-2021. *JAMA Netw Open*. 2024;7(2):e240401. doi:10.1001/jamanetworkopen.2024.0401

5. Deb P, Norton EC. Modeling Health Care Expenditures and Use. *Annu Rev Public Health*. 2018;39(1):489-505. doi:10.1146/annurev-publhealth-040617-013517

© 2025 Cai CL et al. *JAMA Network Open.*
