# Supplementary material for: Use and Costs of Supplemental Benefits in Medicare Advantage, 2017-2021
Source: JAMA Netw Open. 2025 Jan 14;8(1):e2454699. doi: 10.1001/jamanetworkopen.2024.54699 (PMC11733699; doi:10.1001/jamanetworkopen.2024.54699)
Supplement: Supplement 2. — Data Sharing Statement [file jamanetwopen-e2454699-s002.pdf]

Data Sharing Statement

Cai. Use and Costs of Supplemental Benefits in Medicare Advantage, 2017-2021. *JAMA Netw Open*. Published January 14, 2025. doi:10.1001/jamanetworkopen.2024.54699

Data
Data available: Yes
Data types: Deidentified participant data, Data (not involving human participants)
How to access data: Data used for analysis available upon request
When available: With publication

Supporting Documents
Document types: Statistical/analytic code
How to access documents: Statistical /analytical code available upon request
When available: With publication

Additional Information
Who can access the data: Researchers interested in using the data
Types of analyses: For Research
Mechanisms of data availability: With investigator support
